# Quick versus Quantitative: Evaluation of Two Commercial Real-Time PCR Assays for the Detection of Pneumocystis jirovecii from Bronchoalveolar Lavage Fluids

**DOI:** 10.1128/spectrum.01021-23

**Published:** 2023-06-01

**Authors:** Corrie R. Belanger, Kerstin Locher, Billie Velapatino, Philippe J. Dufresne, Eric Eckbo, Marthe Charles

**Affiliations:** a Division of Medical Microbiology, Vancouver Coastal Health, Vancouver, British Columbia, Canada; b Department of Pathology and Laboratory Medicine, University of British Columbia, Vancouver, British Columbia, Canada; c Laboratoire de santé publique du Québec, Institut national de santé publique du Québec, Sainte-Anne-de-Bellevue, Québec, Canada; University of Maryland School of Medicine

**Keywords:** *Pneumocystis* pneumonia, fungal infection, real-time PCR

## Abstract

Two commercial real-time PCR assays for the detection of Pneumocystis jirovecii were compared, the quantitative RealStar P. jirovecii assay and the qualitative DiaSorin P. jirovecii assay, the latter of which can be used without nucleic acid extraction. Archived bronchoalveolar lavage (BAL) specimens (*n* = 66), previously tested by molecular methods, were tested by both assays, and the results were compared to the respective original result. The RealStar P. jirovecii assay demonstrated good positive percent agreement (PPA) (90% [95% confidence interval (CI), 72 to 97%]; 27/30) and negative percent agreement (NPA) (100% [95% CI, 88 to 100%]; 36/36) with the reference method. The DiaSorin P. jirovecii assay concordantly detected P. jirovecii in 19 of 24 positive BAL samples (PPA = 73% [95% CI, 52 to 88%]). All negative BAL samples gave concordant results (NPA = 100% [95% CI, 87 to 100%]; 34/34). Discordant results occurred mostly in samples with low fungal loads. In conclusion, the RealStar assay demonstrated good concordance with reference results, and the DiaSorin P. jirovecii assay performed well for negative BAL and positive BAL samples with P. jirovecii concentrations of greater than 260 copies/mL.

**IMPORTANCE** Pneumonia, caused by the opportunistic fungus Pneumocystis jirovecii, poses a significant risk for immunocompromised individuals. Laboratory testing for P. jirovecii is progressively shifting toward the use of molecular tests such as real-time PCR; however, this is often performed at reference laboratories. Many frontline laboratories are looking into improving their service and reducing turnaround times for obtaining P. jirovecii results by bringing molecular P. jirovecii testing in-house. We evaluated and compared two commercial real-time PCR assays with different workflows for the detection of P. jirovecii from bronchoalveolar lavage specimens. The RealStar P. jirovecii assay requires nucleic acid extraction and provides a quantification of fungal load for positive samples. The DiaSorin P. jirovecii assay offers a simple workflow without nucleic extraction from patient samples and qualitative results. Results from this study provide valuable information on performance and workflow considerations for laboratories that wish to implement P. jirovecii molecular testing.

## INTRODUCTION

Pneumocystis jirovecii pneumonia (PJP) is an opportunistic fungal infection that affects almost exclusively immunocompromised patients. This fungal organism is ubiquitous, with approximately 80% of infants having been exposed by a young age (1–3). It is also estimated to colonize as many as 20% of healthy individuals (4–6). Since the 1980s, there has been a continuous increase in the prevalence of PJP, first described during the onset of the human immunodeficiency virus (HIV) epidemic (7). The organism can cause severe pneumonia, leading to fever, cough, and respiratory distress (8) that can rapidly progress in certain patients. With the advances in HIV treatment and routine prophylaxis for PJP, the frequency of infections in this patient population has decreased significantly (9). However, there has been a notable increase of PJP in individuals receiving immunosuppressive therapy for solid organ or hematopoietic transplants, malignancies, or other inflammatory diseases, and despite PJP prophylaxis, PJP poses a significant risk for these patients (10). Furthermore, nosocomial outbreaks have also been documented, especially in renal transplant cohorts, indicating patient-to-patient transmission of *P. jirovecii* (11, 12).

The organism cannot be routinely cultured and is traditionally identified by microscopy. Conventional staining techniques, such as Grocott-Gomori methenamine silver (GMS), Giemsa stain, or toluidine blue (11, 13), require technical expertise and are relatively insensitive. Immunofluorescent staining has improved sensitivity but can be prone to artifacts that complicate interpretation in patients with low to moderate fungal burden (14). Recently, molecular assays for the detection of *P. jirovecii* have become increasingly common for diagnosing PJP in correlation with clinical symptoms (15–17). PCR assays have been praised for their advantage in detecting the lower burden of *P. jirovecii*, such as in patients with mild to moderate immunosuppression, at concentrations that would be difficult to detect via microscopy methods (16), although this increased sensitivity has led to a debate around the difficulty to distinguish between colonization and infection (18–20). Some groups have proposed real-time PCR cycle threshold (*C_T_*) cutoffs or fungal load thresholds for *P. jirovecii* pneumonia versus colonization, but thus far, no clear guidance has been established (20, 21). As such, definitive PJP infection cutoffs will likely depend on the PCR protocol and the patient population. For example, non-HIV immunocompromised patients present with significantly lower organism concentrations than those seen in HIV patients with PJP.

Both in-house and commercial real-time PCR assays for the detection of *P. jirovecii* using different gene targets have been developed (20, 22, 23). Among *P. jirovecii* real-time PCR assays on the market today are the altona RealStar Pneumocystis jirovecii PCR kit 1.0 (24–26) and the DiaSorin *P. jirovecii* real-time assay (27, 28). Both assays target the multicopy mitochondrial large-subunit rRNA (*mtLSU*) gene (26, 28). The RealStar PJP assay provides quantitative results from extracted nucleic acids based on a standard curve, whereas the DiaSorin assay offers the convenience of real-time PCR detection of *P. jirovecii* directly from respiratory specimens without prior nucleic acid extraction.

To our knowledge, the performance of these two assays has not been compared previously, and our study is the first to compare the DiaSorin assay to another molecular assay. Recent studies have demonstrated the superior sensitivity of the DiaSorin PJP assay when compared to a direct immunofluorescence assay (28) and its clinical usefulness for the diagnosis and management of PJP in correlation with clinical data and pathology testing (27). The RealStar assay has shown excellent concordance with several other molecular PJP assays and has been reported to detect low burdens of *P. jirovecii* in respiratory specimens (24–26).

We evaluated and compared the performance and workflow of the two molecular assays for the detection of *P. jirovecii* in retrospective clinical bronchoalveolar lavage (BAL) specimens.

## RESULTS

### RealStar PJP assay performance.

A total of 66 archived BAL specimens (PJP detected, *n* = 30; PJP not detected, *n* = 36) were available for testing by the RealStar PJP assay for this study (Table 1). Specimens had been previously tested at two different reference laboratories using either a lab developed real-time PCR test (LDT) or the RealStar PJP assay, which were regarded as the reference results.

**Table 1:** Overview of specimens used

Specimen type	Result	Total no.	No. previously tested by:	
			Reference method 1^*a*^	Reference method 2^*b*^
BAL	Positive^*c*^	30	14	16
	Negative	36	3	33

a Reference method 1 is the RealStar PJP assay performed at a reference laboratory.

b Reference method 2 is the LDT PJP assay.

c Positive signal for PJP at any *C_T_* value.

Twenty-seven of 30 PJP-positive BAL specimens were concordantly positive by the RealStar PJP assay in this study, and all 36 PJP-negative specimens gave concordant negative results when compared to the reference method (Table 2). Of the 3 specimens that were discordant, two had low analyte concentrations and were reported as indeterminate by the respective laboratory where they were initially tested (Table 3). Unfortunately, there was insufficient sample volume for discordant analysis for these two samples. The third discordant sample was a mucoid BAL specimen with an original *C_T_* of 27 for the *mtLSU* gene (reference method 2). Upon retesting this specimen by the RealStar assay, using extended Mucolyse treatment, a PJP-positive result was observed with a *C_T_* of 26.6 (43,000 copies/mL).

**Table 2:** Performance of the RealStar PJP assay and DiaSorin PJP assay compared to reference method results

Assay	PJP result	Reference method result		PPA (%)		NPA (%)		Kappa	
		No. positive^*a*^	No. negative	%	95% CI	%	95% CI	Value	95% CI
RealStar PJP Assay	Positive^*a*^	27	0	90	72–97	100	88–100	0.91	0.81–1.0
	Negative	3	36						
DiaSorin PJP Assay	Positive^*a*^	19	0	73	52–88	100	87–100	0.75	0.59–0.92
	Negative	7	34						

a Positive signal for PJP at any *C_T_* value.

**Table 3:** Overview of specimens with discordant results between the RealStar and/or DiaSorin PJP assay and the reference method

Reference method	Reference result (copies/mL)	RealStar PJP assay		DiaSorin PJP assay result
		Result	*C_T_* (copies/mL)	
1	Positive (66)	Negative	n/a	Negative
2	Positive^*a*^	Negative	n/a	Negative
2	Positive^*b*^	Negative^*c*^	n/a^*e*^	n/d^*d*^
1	Positive (21)	Positive	35.2 (125)	Negative
1	Positive (21)	Positive	35.8 (81)	Negative
2	Positive^*a*^	Positive	34.2 (262)	Negative
2	Positive^*a*^	Positive	35.4 (108)	Negative
2	Positive^*a*^	Positive	36.1 (57)	Negative

a Reported as indeterminate by the reference laboratory (*C_T_* results unknown).

b Reference result *C_T_* of 33.8 (*cdc2* gene) and 27.8 (*mtLSU* gene).

c RealStar result was positive for PJP on repeat (PJP *C_T_* 26.6).

d Not done (n/d), specimen was not available for testing on the DiaSorin assay.

e not applicable (n/a), CT value undetermined (negative).

The positive percent agreement (PPA) and negative percent agreement (NPA) for the detection of PJP by the RealStar PJP assay were 90% (95% confidence interval [CI], 72.3 to 97.4) and 100% (95% CI, 88 to 100), respectively. The Cohen’s kappa agreement between the reference result and the RealStar assay was 0.908 (95% CI, 0.81 to 1.0), indicating almost perfect agreement. Where quantitative results were available from the reference method, the fungal load calculated from the RealStar assay results was within 10-fold of the reference result with the majority of them within 1 log_2_. The outliers observed were at concentrations outside of the standard curve (2.5 × 10^3^ to 2.5 × 10^6^ copies/mL specimen), where quantification results may have a higher uncertainty (see Table S1 in the supplemental material).

### DiaSorin PJP assay performance.

Of the 66 BAL specimens tested by the RealStar assay in this study, 60 (reference method positive, *n* = 26; reference method negative, *n* = 34) were available for testing on the DiaSorin *P. jirovecii* real-time PCR assay. All 34 negative specimens gave a concordant negative result on the DiaSorin assay. Of the 26 specimens in which PJP was detected by one of the reference methods, 19 concordant positive results were observed by the DiaSorin assay, and 7 were determined to be negative (Table 2). All 7 discordant specimens had low analyte concentrations with results reported as indeterminate by the respective reference laboratory (Table 3). Unfortunately, discordant analysis was not possible due to low sample volume.

The DiaSorin PJP assay demonstrated a PPA of 73.1% (95% CI, 52 to 88) and NPA of 100% (95% CI, 87 to 100) and a moderate overall agreement with the reference results with a Cohen’s Kappa agreement of 0.75 (95% CI, 0.59 to 0.92).

### Performance and workflow comparison between the two assays.

**FIG 1:**
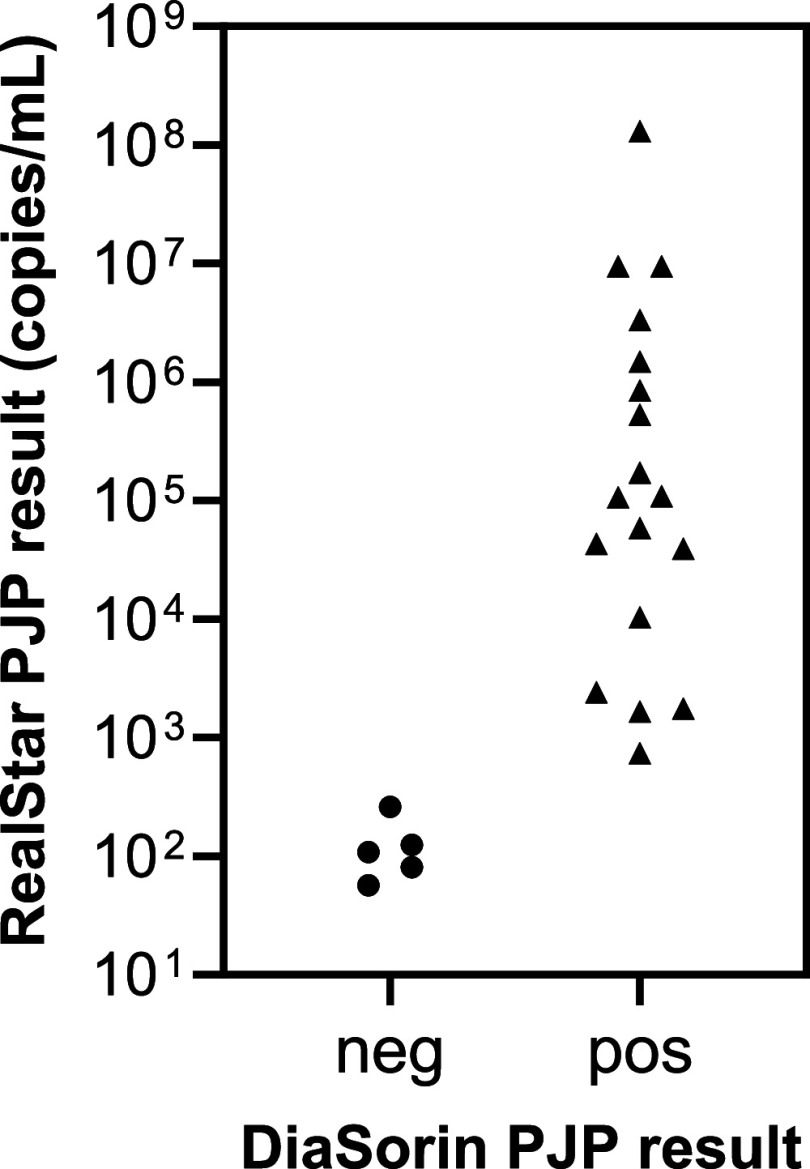
Comparison of RealStar PJP quantitative results and DiaSorin PJP results for 24 BAL specimens that tested positive by at least one assay.

Both assays performed equally well for negative BAL specimens, with 100% agreement between the assays and the reference result. For low positive BAL specimens, the RealStar assay demonstrated a slightly higher positive percent agreement, although this difference was not considered significant (*P* = 0.063 using McNemar’s exact test). On average, the *C_T_*s obtained by the DiaSorin assay in the positive samples were 4.1 higher than the *C_T_*s from the RealStar assay (data not shown). The 7 specimens with PJP-positive results by the reference method and discordant negative results by the DiaSorin assay were either negative (*n* = 2) or had a low PJP concentration (≤262 copies/mL) on the RealStar assay (Fig. 1).

Both the RealStar and the DiaSorin PJP real-time PCR assays were easy to use and required similar hands-on time. The specimen volume needed for the RealStar assay is larger than the DiaSorin assay (200 μL versus 2 μL); however, this is usually not an issue as BAL specimens generally have large volumes. The time to results of the RealStar PJP assay (~180 min) is approximately twice as long as the DiaSorin assay (~70 min), due to the added time for extraction and longer PCR run time.

## DISCUSSION

In this study, we evaluated the performance of two commercial real-time PCR assays, the altona Diagnostics RealStar PJP assay and the DiaSorin PJP assay, for the detection of Pneumocystis jirovecii in bronchoalveolar lavage specimens. The performance of each assay was compared to the original test results from real-time PCR assays performed in different laboratories. Both assays demonstrated very good negative percent agreement (100%) and good concordance for the detection of PJP in BAL specimens that were positive by the reference laboratory. In samples with low organism concentrations (or originally reported as indeterminate), the RealStar assay demonstrated slightly better performance; however, it failed to detect PJP in two BAL specimens with low fungal burden and one mucoid specimen that was positive upon repeat. The reason for the initial false negative result in this specimen is unknown. Generally, the RealStar assay performed well on mucoid specimens when used in combination with a mucolytic agent. A recent study by Scharmann et al. (29) compared the RealStar PJP assay to a commercially available loop-mediated isothermal amplification (LAMP) PJP assay performed on lysed respiratory specimens. This assay, like the DiaSorin assay, does not require nucleic acid extraction, and the authors found that the LAMP assay had a higher limit of detection (LOD) compared to that of the RealStar PJP assay and failed to detect a few positive specimens. In our study, the *C_T_* results from the DiaSorin assay ran on average 3 to 4 *C_T_* values higher when compared to *C_T_* results from the RealStar assay, most likely due to the lack of extraction of specimens. However, The DiaSorin assay reliably detected all BAL specimens with PJP concentrations of more than 260 copies/mL on the RealStar assay. The few positive samples that were missed by the DiaSorin assay were originally reported as indeterminate for PJP by the respective reference laboratory.

The clinical relevance of not detecting PJP in specimens with such low fungal loads is likely low, as these results may not represent acute *P. jirovecii* pneumonia (18, 19, 30). There is much debate about the differentiation between colonization and active disease when using real-time PCR for the detection of PJP, and a consensus on either fungal organism load thresholds or *C_T_* cutoff thresholds to distinguish between colonization and disease has not been established (21). Several studies suggest that real-time PCR results with high *C_T_* values or quantitative results that correlate with low fungal burden makes *P. jirovecii* pneumonia unlikely; however, cutoffs can vary between different assays or patient populations (18, 20, 30–32). Depending on the molecular assay that is being used, each laboratory should establish a cutoff that is reflecting their patient population and pretest probability. Outside of strict PJP diagnostic, it must be noted that it may be useful in certain contexts to detect low-level fungal burden in at-risk populations, such as in the case of nosocomial PJP outbreaks, to check *Pneumocystis* burden prior to transplant or immunosuppression, or as a follow-up to a patient’s response to treatment for *Pneumocystis*.

Our study has a few limitations. Due to the low availability of specimens, some of the specimens used were sourced from a laboratory where they had been tested by the same assay as one of the two methods compared in this study (the RealStar assay), which could have introduced bias. The majority of specimens (74%), however, had been initially tested by a laboratory using a real-time PCR assay (LDT) different from the two assays compared. Clinical information, such as immune status or symptoms of the patient were not available for any of the samples, as collection of this information was outside of the scope of this study. Additionally, quantitative results from reference methods were not available for all of the PJP-positive specimens, and thus, we were not able to corroborate all quantitative results from the RealStar assay. Limited volume of specimens also prevented discordant analysis on most specimens; however, the majority of discordant results were explainable by low organism concentrations in the sample. A few PJP-positive specimens that were tested by the RealStar assay were not available for testing on the DiaSorin assay, which could have impacted the PPA of this assay.

When comparing the workflows of the two commercial assays, the hands-on time was similar for both assays; however, the DiaSorin assay offers a conveniently fast time to result and did not require any additional equipment apart from the small footprint Liason MDX cycler. The RealStar real-time PCR assay requires prior nucleic acid extraction of specimens, which in this study was done using an automated system and thus did not require much additional hands-on time. The extraction step adds additional cost; however, it offers the advantage of removing inhibitory substances such as mucus from specimens. Both assays performed well for the detection of PJP in BAL specimens and demonstrated good concordance with reference molecular test results and between the two assays.

## MATERIALS AND METHODS

### Clinical specimens.

A panel of archived BAL samples, positive or negative for PJP (Table 1), were used for this study.

Since our laboratory did not perform molecular PJP testing at the time of this study, specimens were sourced from two reference laboratories, where the initial clinical testing at the time of collection was performed by one of two different real-time PCR assays. Both assays have been validated for diagnostic used by the respective laboratory.

Reference laboratory 1 (Laboratoire de santé publique du Québec [LSPQ]) provided specimens that were tested by the RealStar Pneumocystis jirovecii real-time PCR kit 1.0 (altona Diagnostics, Hamburg, Germany) with nucleic acids extracted on the eMAG or EasyMAG platform (bioMérieux, Saint-Laurent, Canada). Samples with a positive PJP signal up to a *C_T_* of 35 were reported as positive, and samples with a positive PJP signal of *C_T_* ≥ 35 to 38 were reported as indeterminate or “low burden detected.” Additionally quantitative results in copies per milliliter specimen were provided by this laboratory.

The second reference laboratory (Providence Health Care/Vancouver Coastal Health laboratory, Vancouver, British Columbia) tested specimens using a lab-developed real-time PCR providing qualitative results. The assay targets two genes (*cdc2* and *mtLSU*) with nucleic acid extraction performed on the MagnaPure Compact system (Roche, Laval, Canada) (15). Samples with a positive signal for both gene targets were reported as positive, and samples with a positive signal for only one gene target were reported as indeterminate.

Clinical information was not available for any of the specimens. All specimens were archived at −70°C until the time of retesting in this study.

### Real-time PCR with RealStar Pneumocystis jirovecii PCR kit.

Nucleic acid extraction from respiratory samples was optimized for this study using the MagNa Pure 24 Total NA isolation kit on the automated MagNa Pure 24 extraction system (MP24; Roche, Laval, Canada). The optimized protocol used 200 μL of specimen without any preprocessing, except for mucoid samples, which were combined with an equal volume of Mucolyse sputum digestant (Pro-lab Diagnostics, TX, USA), vortexed vigorously, and incubated at room temperature for 10 min or until liquefied. Prior to extraction, 5 μL of internal control (included in the RealStar PJP PCR kit) and 15 μL of 10× phosphate-buffered saline (PBS) were added to each specimen using the MP24 instrument. Nucleic acids were extracted using the Pathogen 200 3.1 protocol with an eluate volume of 50 μL.

The RealStar Pneumocystis jirovecii PCR kit was used according to manufacturer’s instructions. Briefly, reactions were prepared by combining 5 μL of master A, 15 μL of master B, and 10 μL of eluate and were run on the ABI 7500 Fast real-time PCR system (Applied Biosystems, Waltham, USA) using the manufacturer recommended thermocycling profile. Each run included 4 quantified controls provided by the kit (1 × 10^4^ to 1 × 10^1^ copies/μL), which were used to generate a standard curve and to calculate the organism concentration (in copies/mL) in each specimen.

### Real-time PCR assays with DiaSorin Pneumocystis jirovecii real-time PCR assay.

The DiaSorin *P. jirovecii* real-time PCR assay (DiaSorin Molecular LLC, Cypress, USA) was setup according to manufacturer’s instructions. Briefly, 0.2 μL DiaSorin *P. jirovecii* primer pair was mixed with 4 μL temperature activated (TA) master mix, 0.2 μL Simplexa extraction and amplification control DNA, 0.2 μL Simplexa extraction and amplification control primer pair (DiaSorin), and 3.4 μL nuclease-free water. Subsequently, 8 μL of this reaction mixture was added to each well of a 96-well Simplexa Universal disk (DiaSorin), and 2 μL of unprocessed, neat respiratory specimen was directly added to each well inside a biosafety cabinet. PCRs were performed on the Liaison MDX instrument (DiaSorin) using the manufacturer recommended thermocycling program and the liaison MDX Studio Software for data analysis.

### Interpretation of results.

The results of the RealStar *P. jirovecii* assay and DiaSorin *P. jirovecii* assay were compared to the results of the respective reference method for each sample. For the purpose of data analysis, the archived samples with a positive signal for *P. jirovecii* at any *C_T_* by any of the real-time PCR assays were regarded as positive, even though they may have previously been reported as “indeterminate” by the respective reference laboratory. Due to limited sample volume available, discordant analysis was not possible.

### Statistical analysis.

The positive percent agreement (PPA) and negative percent agreement (NPA) and 95% confidence intervals (CI) between the RealStar *P. jirovecii* assay or the DiaSorin *P. jirovecii* assay and the reference results were calculated. Overall agreement between the RealStar or DiaSorin assay and the reference method was measured by calculating the Cohen’s kappa agreement. Differences between test performances were assessed using McNemar’s exact test. All *P* values were two-sided, and values less than 0.05 were considered statistically significant. Statistical analyses were performed in R v4.1.2 (33) and GraphPad Prism v9.1.0 (34).
