# Supplementary material for: Quick versus Quantitative: Evaluation of Two Commercial Real-Time PCR Assays for the Detection of Pneumocystis jirovecii from Bronchoalveolar Lavage Fluids
Source: Microbiol Spectr. 2023 Jun 1;11(4):e01021-23. doi: 10.1128/spectrum.01021-23 (PMC10434167; doi:10.1128/spectrum.01021-23)
Supplement: Supplemental file 1 — Table S1. Download spectrum.01021-23-s0001.pdf, PDF file, 0.09 MB [file spectrum.01021-23-s0001.pdf]

Table S1. RealStar PJP results for samples with a quantitative PJP reference result available

	PJP concentration (copy/mL specimen)	
**sample ID**	**Reference Result**	**RealStar PJP Assay**
L0-----39001*	21	125
L0-----72001*	21	165
L0-----94001*	57	81
L0-----70001*	66	n/a (negative)
L0-----06001*	225	5
L0-----48001	117,324	109,024
L0-----78001	185,796	173,255
L0-----15001	192,738	106,851
L0-----39001	867,300	531,328
L0-----54001	903,000	847,908
L0-----89001*	2,283,000	9,444,815
L0-----17001*	2,310,000	2,562,680
L0-----61001*	3,363,000	1,480,885
L0-----86001*	26,693,280	3,349,319

* Specimen concentration outside of the standard curve of the assay
